# Towards regional access to medicines: the development of the East African Community pooled procurement mechanism

**DOI:** 10.1080/20523211.2024.2390653

**Published:** 2024-08-21

**Authors:** Koray Parmaksiz, Domina Asingizwe, Alison Kaitesi Gichohi, Stephen Karengera

**Affiliations:** a Erasmus School of Health Policy & Management, Erasmus University Rotterdam, Rotterdam, The Netherlands; b EAC Regional Centre of Excellence for Vaccines, Immunization, and Health Supply Chain Management, College of Medicine and Health Sciences, University of Rwanda, Kigali, Rwanda; c Health Department, EAC Secretariat, Arusha, United Republic of Tanzania

**Keywords:** Pooled procurement, joint procurement, bulk purchasing, centralised procurement, pooled procurement guidance, East African Community, EAC, pharmaceuticals, medicines

## Abstract

**Introduction:**

The East African Community (EAC) has been facing challenges in ensuring access to affordable and quality-assured medicines. To address these problems, the EAC Partner States have been working on implementing an inter-country pooled procurement mechanism since 2005. However, with limited progress to date. The aims of this study were to explore how the EAC pooled procurement mechanism has been developing over time, and to clarify the work and efforts made during this development process to draw lessons for enhancing such collaborative efforts.

**Methods:**

For this study, we carried out a multi-method qualitative case study. We used the Pooled Procurement Guidance to collect and structure our data drawn from academic papers, grey literature documents, observations and field notes. For the analysis, we used an inductive thematic analysis approach.

**Results:**

Over the past two decades of the EAC’s pooled procurement journey**,** we have identified two developmental stages so far: the promise stage and the creation stage. The promise stage was characterised by initial engagement and alignment efforts between Partner States. However, the lack of dedicated funding and ownership to drive the project forward led to stagnation of the process for some years. Following the establishment of a dedicated organisation, the pooled procurement mechanism entered the creation stage. This stage has been characterised by continuous alignment work consisting of project management, efforts to build inter-personal relationships, and facilitation of negotiations to harmonise goals, needs and operations. This process has been aided by broad and recurring involvement of regional experts.

**Conclusion:**

To successfully implement a pooled procurement mechanism, we suggest EAC Partner States to continue their alignment efforts, sustain political will and allocate sustainable funding using a phased implementation approach towards pooled procurement.

## Introduction

Medicines are fundamental for managing disease burden. Many low- and middle-income countries, however, face challenges in ensuring access to affordable and quality-assured medicines (Bigdeli et al., 2013; Cameron et al., 2011; Ozawa et al., 2019). Similar challenges have been experienced in the East African Community (EAC); an intergovernmental organisation established to promote economic, political, social and cultural integration. The EAC currently consists of eight countries, referred to as Partner States: The Republic of Burundi, the Democratic Republic of the Congo, the Republic of Kenya, the Republic of Rwanda, the Republic of South Sudan, the Republic of Uganda, the United Republic of Tanzania and the Federal Republic of Somalia. Most of these Partner States have been facing problems to ensure access to quality and affordable medicines. Several factors underly this problem, such as a relatively small market size leading to high prices, inadequate supplier incentives causing shortages of certain essential medicines, insufficient technical capacity or staff resulting in the entry of uncertain or poor quality medicines into the market, limited local production capacity, and inefficiencies in procurement and supply chain processes (EAC RCE-VIHSCM et al., 2019; East African Community, 2018a; Yenet et al., 2023).

In response to these problems, the EAC Partner States started discussions on pooled procurement in 2005 during the 1st EAC Sectoral Council of the Ministers of Health meeting (East African Community, 2005). Pooled procurement can be defined as ‘a collaboration initiative that consists of two or more buyers, or a third-party organization that procures on behalf of its participating members' (Parmaksiz et al., 2022). Setting up such mechanisms is not a standardised process. They differ in structural form (ranging from inter-buyer to third-party), operational level (i.e. local to global), product types (e.g. patented products, disease-specific products, vaccines or a basket of essential medicines), and need to be embedded within the local contextual environment (Parmaksiz et al., 2022). They also differ in levels of collaboration and integration, ranging from *informed buying* (information sharing on prices, product quality and suppliers), *coordinated informed buying* (conducting joint market research), *group contracting* (engaging in joint negotiation) to *central contracting* (through a dedicated procurement agent acting on behalf of the buyers) (Management Science for Health, 2012; World Health Organization, 2020). Additionally, they have a tendency to evolve over time following a path of developmental stages (Parmaksiz, Bal, et al., 2023; Schotanus et al., 2011).

A recent study (Parmaksiz, Bal, et al., 2023) developed a *Pooled Procurement Guidance*, which we will use as an analytical tool to structure and analyse our data. The guidance was developed to assist policy-makers and procurement experts in the implementation and operation of such mechanisms. It was constructed by integrating empirical (i.e. academic and grey literature studies), practical (i.e. expert opinion) and theoretical (i.e. collaborative governance and organisational life cycle literature) insights. It describes how pooled procurement mechanisms tend to develop over time, and which elements are essential in setting up and sustaining such mechanism. A follow-up study (Parmaksiz, van de Bovenkamp, et al., 2023) applied this Pooled Procurement Guidance to both inter-buyer and third-party pooled procurement mechanisms. One of its main conclusions was that establishing inter-country, buyer’s owned pooled procurement mechanisms in practice seems more complex compared to third-party, disease-specific pooled procurement organisations (e.g. Global Fund, Global Drug Facility). This is predominantly a result of diverging procurement, supply chain and financing processes among buyers, along with differing product needs and incompatible legislative frameworks. These heterogeneities add further layers of complexity to the alignment of interests and operations between buyers within inter-buyer mechanisms (Parmaksiz, van de Bovenkamp, et al., 2023).

The EAC pooled procurement mechanism provides another good example of complex, inter-buyer alignment processes. This is best illustrated with the fact that discussions on implementing pooled procurement have been ongoing for nearly two decades. Yet, little is known about how the EAC pooled procurement mechanism has evolved over time. What work and efforts have been made to align interests and develop the mechanism? What challenges and complexities have been encountered? And which lessons have been learned? This paper aims to provide answers to these questions. We believe this longitudinal and in-depth approach is valuable as it increases our understanding of how inter-country, buyer’s owned pooled procurement mechanisms develop in practice. These insights also provide important lessons for other, inter-country pooled procurement mechanisms facing similar challenges.

## Methods

### Research context

Cooperation between East African countries such as Kenya, Uganda and Tanzania dates back to the early 1900s. In 1917, Kenya and Uganda formed a Customs Union, which Tanganyika (now the United Republic of Tanzania) joined in 1927. Between 1948 and 1961 these three countries formed the East African High Commission, which was renamed after independence of the countries into East African Common Services Organisation between 1961 and 1967, and later into the East African Community (EAC). This first iteration of the EAC collapsed after political disagreement between Partner States, and was eventually dissolved in 1977 (Cooksey, 2016; EAC History, n.d.). It was not until 1999 that these three countries formalised their international cooperation with a treaty (East African Community, 2002) by reviving the EAC. Kenya, Uganda and Tanzania became the three founding Partner States of the EAC, who were joined by Rwanda and Burundi in 2007, South Sudan in 2016, and the Democratic Republic of Congo in 2022 (EAC Quick Facts, n.d.). The Federal Republic of Somalia, the latest Partner State, joined in 2023 (East African Community, 2023d). The Democratic Republic of the Congo and the Federal Republic of Somalia, having both recently joined the EAC, have not been actively involved in the development of the pooled procurement mechanism so far. Therefore, their participation and efforts have not been incorporated into this paper.

The EAC is one of the eight Regional Economic Communities (RECs) recognised by the African Union, has a combined population of over 330 million people and its EAC Partner States share a common history, culture and language (Leonard A. Kamwanja et al., 2010). Coordinated by the EAC Secretariat, the EAC is governed by different bodies such as the Summit, the Council of Ministers, East African Legislative Assembly and East African Court of Justice (*EAC Organs,* n.d.). A more detailed description of the EAC bodies, their organisational structure and responsibilities have been included in Supplemental Material 1.

### Study design

We carried out a multi-method qualitative case study. The study consisted of two phases that took place simultaneously, which allowed us to triangulate our data. This study design enabled us to examine how the EAC pooled procurement mechanism has evolved over time, clarify the work and contributions made by different actors, and identify the current challenges, needs, practices and processes (e.g. procurement, financing, regulation) within the EAC Partner States.

The first phase aimed to capture data on how the implementation of EAC pooled procurement mechanism has been developing over time. First, we began by searching academic literature related to the pooled procurement in the EAC in February 2020 in databases such as PubMed and Google Scholar with the following search terms: pooled; joint; bulk combined with procurement or purchasing. These search terms were combined with EAC and East African Community. We used Boolean operators to combine these search terms, and did not limit our search to a timespan to capture as many publications as possible. We found no papers published in academic journals focusing on this subject. Next, we expanded our scope to grey literature documents, including policy papers, feasibility studies, academic theses and newspaper articles. We used the same search terms and looked in databases including Google Scholar, WHO’s Institutional Repository for Information Sharing and the EAC Information Resource Centre. We also scanned reference lists for further publications (i.e. snowballing). Over the years, we continuously updated our search using the same search terms. Additionally, we sought recommendations from key actors in the field for further relevant publications. This process continued until our final search was conducted in April 2024. These searches yielded 8 key documents. In addition, we included 23 meeting reports of the EAC Sectoral Council of Ministers of Health between July 2005 (1st ordinary meeting) and February 2023 (23rd ordinary meeting), and 9 EAC regional stakeholder meeting reports between 2008 and 2023.

The second phase aimed to identify the work and efforts made, and by which actors, during this implementation process. We drew upon unstructured observations of which extensive field notes were made during four regional stakeholder meetings that we were present at between March 2020 and August 2023, with a total duration of approximately 100 h (see Table 1). Our observations were focused on capturing the interaction between stakeholders. Particularly examining how they aligned their respective challenges and needs and negotiated towards a unified pooled procurement mechanism. We wrote down our observations and thick descriptions as fieldnotes, and compiled them into trip reports after each day or after the meeting had been concluded (Emerson et al., 2011; Wolfinger, 2002). In addition, we conducted informal interviews with stakeholders during these regional stakeholder meetings to verify our observations and gather additional background information.

**Table 1.** Overview of stakeholder meetings during which observations took place.

Meeting	Date	Place
Regional Stakeholders’ Meeting to Build Consensus on the Pooled Procurement Model for the EAC Partner States	4–6 March, 2020	Nairobi, Kenya
Regional Meeting to Develop a Detailed Model and Operational Plan for Pooled Procurement	21–25 June, 2021	Nairobi, Kenya
Regional Meeting to Validate the EAC Pooled Procurement Market Survey Report and Model	13–15 March, 2023	Entebbe, Uganda
Meeting of Heads/CEOs of the National (Central) Medical Stores	7–8 August, 2023	Kigali, Rwanda

Data were gathered by researchers and supply chain and procurement experts who have been actively involved at various stages during the development process of the EAC pooled procurement mechanism in activities such as organising and facilitating stakeholder meetings, commissioning reports, next to collecting data. In addition to facilitating data access and collection, our active involvement also allowed us to verify and triangulate our findings among researchers who were engaged in the process. This variation in time of participation is also reflected in the detail presented in our analysis. While some of our data comes from informal interviews with experts involved during the early stages of the EAC pooled procurement mechanism, most of the data on that period is based on document analysis and literature review. As a result, our main focus during this stage was on reconstructing the developmental process. After 2016, when personal involvement of our research team increased, data sources were expanded with personal observations, experiences and data collected for the purpose of the *East African Community Pooled Procurement of Medicines and Health Commodities Market Survey Report* (EAC RCE-VIHSCM et al., 2023), carried out between November 2022 and February 2023. This has resulted in richer analysis of the development of the mechanism and the type of work that took place.

### Data analysis

We used the *Pooled Procurement Guidance* document (Parmaksiz, Bal, et al., 2023) as introduced earlier to structure our analysis through inductive thematic analysis. The Pooled Procurement Guidance consists of two parts. Part 1 identifies essential elements to consider for developing a pooled procurement mechanism, while Part 2 focuses on the development of such mechanisms over time.

In Part 1, essential elements are organised around key actors (i.e. buyers, pooled procurement organisation and suppliers) in the mechanism. These elements can be seen as preconditions or objectives to achieve during the implementation and operation process. They include – but are not restricted to – the presence of compatible laws and regulations, sufficient budget to procure health products and cover organisational expenses, sufficient technical capacity for accurate demand forecasting, regulatory harmonisation, trust, transparent information sharing, and sufficient incentives for suppliers to participate in the mechanism.

In Part 2, four general developmental stages have been identified: the promise stage, the creation stage, the early operational stage and the mature stage. This classification enabled us to delineate the developmental stages that the EAC pooled procurement mechanism has gone through so far: the promise stage and the creation stage. We then carried out a more systematic analysis of all EAC Sectoral Council of Ministers of Health meeting reports and EAC regional stakeholder meeting reports to identify key events and trace its chronological development over time, using NVivo (12.7.0) as the qualitative data analysis software.

Next, we carried out a thematic synthesis analysis to explore which factors influenced those key events within each developmental stage. The essential elements in Part 1 of the *Pooled Procurement Guidance* document were used as an analytical tool to structure and analyse the most important themes emerging from our collected data (i.e. literature review, document analysis, observations, informal interviews). A comprehensive review of the EAC pooled procurement mechanism with supporting references using the Pooled Procurement Guidance document has been included as an appendix (see Supplemental Material 1).

We then discussed our findings within the research team during multiple sessions until we reached consensus on the analytic themes. After refining our descriptive themes, we arrived at the following set of analytical themes that together provide insights into the historical development process of the EAC pooled procurement mechanism: (1) initial stakeholder engagement; (2) seeking alignment; (3) presence/absence of process ownership and accountability; (4) broad involvement and continuity in representation; (5) project management; and (6) reflexive process and ongoing negotiations.

## Results

**Figure 1.**
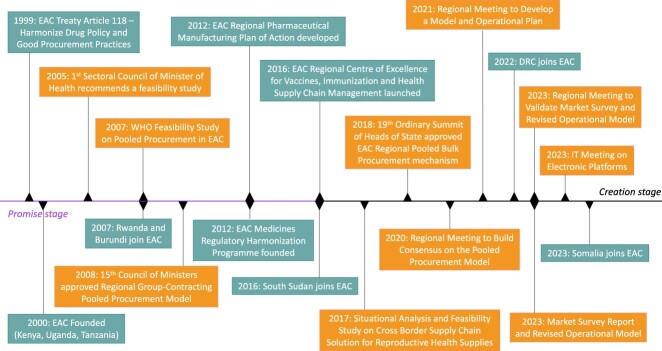
Timeline of key developmental events of the EAC pooled procurement mechanism. Grey blocks refer to general developments in the EAC; orange blocks refer to developments related to the EAC pooled procurement mechanism.

In this section, we present our findings on the developmental process of the East African Community (EAC) pooled procurement mechanisms. Based on the classification of Parmaksiz, van de Bovenkamp, et al. (2023), we have organised this section around two developmental stages: the promise stage (1999–2015) and the creation stage (2016–present) (Figure 1).

### Promise stage

The promise stage is characterised by creating engagement between potential buyers, while they try to convert their perceived problem(s) or opportunities into a shared vision (Parmaksiz, Bal, et al., 2023). In this section, we will focus on three themes that emerged from our data analysis of our literature review and informal interviews: (1) initial stakeholder engagement, (2) seeking alignment, and (3) designating ownership.

#### Initial stakeholder engagement

The foundations for the discussions around pooled procurement in the EAC region were laid with the re-establishment of the EAC. Article 118 of the Treaty for the Establishment of the EAC (East African Community, 2002) focuses on increased cooperation in terms of developing ‘a common drug policy which would include establishing quality control capacities and good procurement practices' (Article 118c) and ‘harmonise drug registration procedures so as to achieve good control of pharmaceutical standards without impeding or obstructing the movement of pharmaceutical products within the Community' (Article 118d).

It took, however, a few more years before pooled procurement came on the EAC agenda. In 2005, during the 1st Ordinary Meeting of the Sectoral Council of the Ministers of Health (OM-SCMoH) (East African Community, 2005), the health ministers recommended to carry out a situational analysis on ‘medicines policy, legal and regulatory framework, procurement, distribution and management within the EAC Partner States to facilitate the process of harmonisation within the EAC.' The ministers pointed out that within this study, special attention should be paid to learn from experiences of already existing inter-country pooled procurement mechanisms, and the possibilities and challenges to set up an inter-country pooled procurement mechanism for selected essential medicines, including HIV/AIDS, malaria and tuberculosis medicines (East African Community, 2005). Discussions continued in November 2006, when regional stakeholders came together in Zanzibar during a meeting on pooled procurement of ARVs and Essential Medicines in the EAC. During this meeting, stakeholders re-iterated their interest to implement an inter-country pooled procurement mechanism. During a regional expert meeting in Arusha, Tanzania in April 2007, the experts proposed to analyse the feasibility of *group contracting* and *central contracting* as options for pooled procurement in the EAC (East African Community, 2007; WHO et al., 2007).

#### Seeking alignment

After these initial engagement efforts between regional stakeholders, EAC Partner States took their first steps in aligning interests. They started with a situational analysis and needs assessment of the countries with the intention to converge them into common goals and purposes for the pooled procurement mechanism.

In 2007, a comprehensive assessment looked at political and organisational commitment, procurement legislations and policies, medicines regulatory procedures, medicine supply chain systems, financial resources & systems, and pricing policies. Based on their findings, the consultants suggested that group contracting would be more feasible for the EAC than central contracting (WHO et al., 2007). During the 2nd OM-SCMoH in 2007 in Arusha, Tanzania, the health ministers considered and approved the final report and recommendations of the situational analysis (East African Community, 2007). Six months later, during the 15th Ordinary Meeting of the Council of Ministers in March 2008 (East African Community, 2008; Syam, 2014), the central decision-making and governing organ of the EAC consisting of Ministers or Cabinet Secretaries from the Partner States responsible for regional co-operation, approved the group contracting as the pooled procurement model for the region.

In September 2008, the 3rd OM-SCMoH (East African Community, 2008) held in Entebbe, Uganda, directed the EAC Secretariat to develop a draft strategic plan of action and multi-year budget for the implementation of such a mechanism. In addition, the health ministers appointed a wide variety of stakeholders from the Partner States to the *Regional Essential Medicines Pooled Bulk Procurement Expert Task Force*, and urged Partner States to establish a national task force in each country to facilitate coordination between the national and the EAC level.

In September 2009, stakeholders convened during a regional meeting on *Regional Harmonisation of Policies, Legal and Regulatory Framework for EAC Partners States National Medicines Regulatory Authorities (NMRAs) and National Medicines Procurement Agencies (NMPAs)* (East African Community, 2009). During plenary discussions, the stakeholders agreed to tackle potential issues of resistance to change among NMPAs, to develop a regional Essential Medicine List, to prioritise activities to ensure progress, and to update the members of the Expert Task Force.

Up to this point, the Partner States had been showing clear and sustained high-level political commitment to explore the feasibility of pooled procurement for health products in the region. This commitment was visible on multiple actor levels, including Ministers of Health, EAC council of Ministers and regional experts from Partner States.

#### Designating ownership

However, limited progress was made in the following years. During the 5th OM-SCMoH in 2011 (East African Community, 2011a), the health ministers reiterated their directive to the EAC Secretariat to finalise the development of a business plan and prepare the Terms of Reference for the National Task Forces in the EAC Partner States. The topic of pooled procurement was not discussed during three consecutive OM-SCMoH (East African Community, 2011b, 2012a, 2012b).

One of the major issues at that time was the lack of dedicated funding from EAC Partner States. Although the Health Department of the EAC Secretariat kept oversight and organised meetings on pooled procurement, they were responsible for a wide variety of tasks on numerous health-related topics. Therefore, the project lacked a designated driving force and a system in which organisations could be held accountable for delays in the process. To stimulate progress and increase ownership of various priority areas in the region, the EAC Secretariat proposed assigning specific Partner States to host certain projects. During the 9^th^ OM-SCMoH in April 2014 (East African Community, 2014), the EAC Sectoral Council of Ministers of Health directed the EAC Secretariat to set up Regional Centres of Excellence (RCoEs, now RCE) for **‘**skills and tertiary education in higher medical and health sciences education program' across the region, both in terms of research and healthcare delivery. Each RCE would be hosted by one of the EAC Partner States. The Republic of Rwanda was assigned to host the *EAC Regional Centre of Excellence for Vaccines, Immunization and Health Supply Chain Management* (EAC RCE-VIHSCM) among others.

One of the EAC RCE-VIHSCM’s core responsibilities would be to preserve the progress of the pooled procurement initiative (East African Community, 2014). This development also marks the end of the promise stage, in which buyers were mainly concerned with converting their individually perceived problem(s) or opportunities into a shared vision through stakeholder engagement, regular meetings, situational analyses and feasibility studies (Parmaksiz, Bal, et al., 2023).

### Creation stage

The creation stage is characterised by the joint efforts of stakeholders to build consensus around goals and needs, develop a shared operational plan and mobilise sufficient resources (Parmaksiz, Bal, et al., 2023). In this section, we will focus on four themes that emerged from our data analysis of our literature review, observations and field notes: (1) process ownership and accountability, (2) broad involvement and continuity in representation, (3) project management, and (4) reflexive process and ongoing negotiations.

#### Process ownership and accountability

The mechanism’s transition from the promise stage to the creation stage was propelled by the establishment of the EAC Regional Centre of Excellence for Vaccines, Immunization, and Health Supply Chain Management (EAC RCE-VIHSCM). In March 2016, the EAC RCE-VIHSCM, hosted by the University of Rwanda was launched with financial support from Germany’s Federal Ministry for Economic Cooperation and Development (BMZ) through the German Development Bank (KfW), which has since been its biggest funder. One of the projects that EAC RCE-VIHSCM was mandated to facilitate, together with the EAC Secretariat, was establishing the inter-country pooled procurement of health commodities (EAC RCE-VIHSCM, 2019; East African Community, 2019b). The EAC RCE-VIHSCM’s embedding and close collaboration with the EAC Secretariat legitimised their operations and expedited the trust-building process among key stakeholders from various Partner States.

After setting up their organisational structure and hiring skilled staff to run the centre, the EAC RCE-VIHSCM started the pooled procurement project with conducting a feasibility study and situational analysis on cross-border supply chain solutions for reproductive health in 2017 (IMS Health, 2017).

The study's inception report was approved in May 2017 (East African Community, 2017), and the final report was validated in October 2017 after consultations with five out of six Partner States due to national elections in Kenya. The 16^th^ OM-SCMoH in 2018 (East African Community, 2018b) acknowledged the progress made, but was dissatisfied with the study's conduct, directed finalising the in-country validation process and resubmitting the feasibility study. The country visit to Kenya took place in December 2018, a couple days before the next regional stakeholders meeting. At this regional stakeholder meeting in 2018 in Naivasha, Kenya (East African Community, 2018c), stakeholders recommended to set up an adequately staffed pooled procurement secretariat, secure sufficient financial resources, and develop harmonised procurement policies. They also agreed to integrate lessons from the feasibility study on reproductive health into the ongoing essential health commodities project to avoid duplication (East African Community, 2018c).

In the following year in 2019, the Sectoral Council of Ministers of Health took note of the recommendations and directed the EAC Secretariat and the EAC RCE-VIHSCM to conduct a regional meeting to build consensus on the inter-country pooled procurement model before the 19^th^ Ordinary Sectoral Council meeting to be held later that year (East African Community, 2019a). This deadline could not be met, and it was not until 2020 that this meeting was held in Nairobi, Kenya.

The findings show us that the presence of a dedicated organisation or secretariat that is equipped with ample resources and mandate was crucial for the development of this mechanism. The establishment of the EAC RCE-VIHSCM gave new impetus to the alignment process, which can be observed by the increased frequency of regional stakeholder meetings between Partner States in the following years. The EAC RCE-VIHSCM’s institutional backing by and strong collaboration with the EAC Secretariat further legitimised their operations. In addition, our findings showed that discussions between Partner States during those frequent meetings focused on laying the groundwork for shaping the initial structure of the mechanism.

#### Broad involvement and continuity in representation

In March 2020, the EAC Secretariat in collaboration with the EAC RCE-VIHSCM organised the *Regional Stakeholders Meeting to Build Consensus on the Pooled Procurement Model for the EAC Partner States* (East African Community, 2020). A wide variety of stakeholders from all participating EAC Partner States were present. In addition, representatives from global health agencies such as WHO, WHO-Afro and USAID were present to share their experiences with inter-country pooled procurement mechanisms. The meeting was facilitated by an external consultant, who presented best practices from existing pooled procurement mechanisms and a brief situational analysis of the Partner States. After plenary sessions, the representatives from the EAC Partner States were divided into working groups to discuss and provide input on topics such as country preparedness towards inter-country pooled procurement, availability of resources for information sharing and work sharing. Additional working groups sessions were held on budgeting and funding, current laws and regulations, developing a roadmap and structural arrangements for inter-country pooled procurement mechanism. These working group sessions were followed by plenary discussions with all representatives. Discussions evolved around the proposed governance structure of the mechanism. Some international global health organisations suggested a more efficient and leaner structure that operates more independently from national structures. Although the representatives agreed with the suggestions of a lean structure, they emphasised the hierarchical nature of operations within the EAC. The pooled procurement governance structure had to be integrated into the overall EAC governance structure, since working groups and committees are guided by higher-level council decisions and directives.

Another example in which the hierarchical nature of operations in the EAC became apparent was during discussions on the pace of implementation:

It was the third and final day of consecutive meetings. The stakeholders, visibly tired from the discussions of the previous days, had to address one final pressing issue on the implementation approach before the meeting report could be drafted.

The consultant proposed to conduct a phased implementation approach to pooled procurement, starting with information sharing. While two Partner States initially agreed with the suggested approach of the consultant, two other Partner States disagreed by putting forward the Sectoral Council of Ministers of Health’s directive to implement a group contracting model. Therefore, the focus of this meeting should not be on information sharing but on group contracting. These Partner States were mostly concerned about further possible delays of the implementation process. With the guidance of the consultant, the Partner States eventually reached consensus on a phased approach. The Partner States recognized that these two phases of pooled procurement are not mutually exclusive. In fact, information sharing is an important precondition to reach group contracting. [Fieldnotes Nairobi meeting March 2020]

The meeting concluded with recommendations to leverage existing expert knowledge on procurement within the region and put in place a mechanism to share this expertise among Partner States.

After the 2020 Nairobi meeting, the EAC Secretariat and the EAC RCE-VIHSCM organised another regional stakeholder meeting in June 2021 with the goal to *Develop a Detailed Model and Operational Plan for Pooled Procurement* in Nairobi, Kenya (East African Community, 2021). Continuity in representation turned out to be an important facilitator in the trust-building process among stakeholders. The facilitator, many of the country delegates and supporting staff had been involved in previous meetings on pooled procurement in the region. This expedited the familiarisation process between stakeholder, resulting in open and transparent discussions during plenary discussions.

During this meeting stakeholders held discussions on the goals and needs of the pooled procurement mechanism. The vision, mission, guiding pillars and strategic objectives were all formulated, reviewed and agreed upon in a plenary session. The needs for the products to be procured were not agreed upon during this meeting. Although the Partner States reached consensus on including essential medicines and not focus on products that are procured from or by global health partners in the early stages of the mechanism, the specific types of products were yet to be determined. Since priorities for health products might differ for each Partner State, the Partner States agreed to submit their top categories of health commodities at a later stage.

When stakeholders are unable to align interests, we observed that sometimes, an external actor might be needed to bring stakeholders together when discussing sensitive topics, as seen in the following example on the governance structure:

The contrast with previous year’s regional stakeholder meeting could not have been bigger: this time with masked participants, socially distanced chairs, and even dedicated hotel staff tasked with sanitizing microphones.

After lively discussions on the potential governance structure previous year, we (i.e. the facilitators) left the topic of the governance structure for last because we did not want it to negatively influence discussions on other subjects. Initially, agreeing on a governance structure proved challenging. Partly because of the plenary setup of the session, which made developing a focused structure difficult, and partly because each Partner State wanted to remain in control over their procurement processes. This meant that the Partner States expected to be part of the decision-making process in the pooled procurement mechanism.

After plenary discussions reached an impasse, one of the stakeholders proposed to ask the facilitator to draft a governance structure based on lessons from other inter-country pooled procurement mechanisms. The stakeholders agreed and the meeting was adjourned for an hour. As the facilitator of this meeting, KP initially felt a bit at unease with this new dual role as consultant and researcher. He had to step in last-minute because last year’s facilitator could not attend this meeting due to COVID-19 restrictions and his replacement got rejected a visa before boarding. Nevertheless, he agreed with the task, found a quiet spot, came up with a draft governance structure based on existing inter-country pooled procurement mechanisms while taking the recommendations of the 2020 Nairobi meeting into account, discussed them with his colleague consultants and presented it after the plenary session resumed. After minor changes, the stakeholders agreed on the governance structure proposed by the facilitator and discussions continued onto the next subject. [Fieldnotes Nairobi meeting June 2021]

The next subject to be discussed was on increasing ownership of the project at the national level. The stakeholders recommended to nominate a focal person from each Partner State who would be responsible for communication, monitoring progress and data collection within each country. Also, the focal person would collaborate closely with the EAC Secretariat, EAC RCE-VIHSCM and the consultants to drive the implementation process forward. This was another example of ownership and responsibility of the project, but on the Partner State (i.e. national) level.

After the plenary sessions, technical experts were divided into three groups to discuss conditions for successful information sharing, the information to be shared, and the sharing platform. Although this exercise identified important factors, experts agreed that a more in-depth market survey was needed. In the final plenary discussion, Partner States agreed the survey should assess each Partner State's procurement processes, financial mechanisms, legislation, technical capacity, market size, and local production capacity for a phased approach towards group contracting. The goal was to complete the market survey and present it for approval during the 21st OM-SCMoH later that year.

These examples show that continuity in representation facilitates trust-building and strengthening of personal relationships between stakeholders. In turn, these relationships promote open and transparent communication during regional meetings. In addition, calling in externals is a deliberate and effective strategy deployed to navigate impasses around sensitive topics. The combination of these factors turned out to be an essential precondition for aligning interests, goals and purposes.

#### Managing the project

Managing the project in practice, however, does not always go according to plan. Preparations for the market survey analysis started in July 2021 with the development of the Terms of Reference by the EAC Secretariat in collaboration with the EAC RCE-VIHSCM and experts from the EAC Partner States for the assignment. After initial agreement with two consultants, and a supporting role for KP (i.e., the first author), the consultants and the EAC RCE-VIHSCM agreed that conducting a comprehensive analysis could not be completed before the next Sectoral Council of Ministers of Health meeting planned in December later that year. Therefore, they decided to carry out the market analysis over a longer period of time. In October 2021, a new consultant was hired because the previous consultants were not available anymore. After initial meetings on the setup of the market survey analysis between the EAC RCE-VIHSCM, the consultant and the focal persons in early 2022, there were delays in obtaining official requests for collecting data from some of the Partner States for the analysis.

In September 2022, the task resumed again with the consultant and KP conducting a background study, developing data collection tools and an inception report with input from EAC RCE-VIHSCM and the focal persons. These were approved during an online meeting with regional stakeholders in December 2022. Around the same time, data collection through questionnaires and semi-structured interviews started. The focal persons were instrumental during this process. They were responsible for distributing the questionnaires to local experts and collecting data within the Partner States, and providing additional input during the analysis of the data. This was an iterative and reflexive process. After three rounds of meetings with each focal person and three group meetings, the consultant and KP finalised the draft of the market survey report.

This example shows us that initial plans often deviate from practice. Many factors, both related or external to the specific project, can contribute to this. For instance, a staff member might be replaced or an emergency situation such as a pandemic or a critical shortage of medicines might occur that demands time and resources, resulting in delays of the ongoing project. A clear ownership of the project in combination with sufficient resources is therefore required. Not only to keep momentum going, but also to overcome such obstacles.

#### Reflexive process and ongoing negotiations

The output of the market survey analysis was presented during a regional stakeholder validation meeting in Entebbe, Uganda in March 2023 (East African Community, 2023b). Continuity in representation was also visible during this meeting. Most of the country delegates in Entebbe were also present during the 2020 and 2021 regional stakeholder meetings in Nairobi. One of the things that became obvious during these meetings was the continuous and reflexive nature of the alignment process between stakeholders. Although some of the goals and needs were already discussed during previous meetings, organising these stakeholder meetings allowed Partner States to revise and re-negotiate previous decisions. One example was the selection of products to be procured. During both Nairobi meetings in 2020 and 2021, the stakeholders agreed to start information sharing on essential medicines, which are often required in high volumes, are needed by all Partner States, and have already obtained market access in each Partner State. However, during the Entebbe meeting, some Partner States highlighted the need to include regionally produced pharmaceuticals to incentivise local manufacturing, while other Partner States emphasised the need to include ‘hard to source' products in the joint products list. Based on these suggestions, this meeting recommended that the EAC Secretariat should send out a letter to each Partner State requesting a list of hard to source products to be included in the initial list of products for information sharing. Currently, the EAC Secretariat is in the process of collecting these hard to source product lists.

Another point of discussion during stakeholder mapping was that buy-in was required from various stakeholders such as Central Medical Stores to avoid friction and slow implementation. Therefore, the meeting recommended to present the EAC pooled procurement framework and revised Operationalization Model to the heads of National/Central Medical Stores and relevant experts, including Chief Pharmacists and Ministry of Health officials.

Following the Entebbe Meeting in March 2023, the EAC Secretariat in collaboration with the EAC RCE-VIHSCM started the implementation of the meeting recommendations. In May 2023, a meeting was organised to develop the technical specifications for the digital platform that will facilitate information sharing, and eventually pooled procurement (East African Community, 2023c). During this meeting, stakeholders discussed various desired modules for information sharing and agreed on the frequency and initial set of activities/information to be shared among the Partner States. These include stock status, availability and registration status of selected health commodities. In addition, Partner States agreed to share information on local and foreign manufacturers.

In August 2023, the EAC Secretariat in collaboration with the EAC RCE-VIHSCM organised a regional stakeholder meeting in Kigali, Rwanda with the CEOs of the National/Central Medical Stores from each Partner State, as suggested by the regional stakeholders in the Entebbe meeting five months earlier (East African Community, 2023a). During this meeting, key findings of the market survey report, the proposed governance structures during information sharing (Phase 1) and group contracting (Phase 2) and the information to be shared were presented by the EAC RCE-VIHSCM. The aim was to inform the Central Medical Stores, receive their input, and obtain their buy-in and approval as key stakeholders in the pooled procurement mechanism.

The reflexive and continuous nature of the negotiations were also seen at this meeting, where discussions on the initial list of health products for information sharing continued:

Around 40 participants were present during the 2-day regional stakeholder meeting, including CEOs of the National/Central Medical Stores, chief pharmacists and Ministry of Health officials. The participants had been listening to three back-to-back presentations on the progress made so far on pooled procurement in the region. After the final presentation, the floor opened for a plenary discussion. One of the stakeholders took the opportunity and proposed to go for lunch first but to no avail. He got opposed by his colleagues. One of them replied: we should continue and forge the iron while it is still hot.

The stakeholders proceeded to discuss the initial list of health products for information sharing at launch. A representative from one of the Partner States commented that the list should reflect the products that they, as a country, have difficulties procuring. A representative from another Partner State agreed and added that the list should also include more products on non-communicable diseases. A third representative disagreed and commented that the list should not be overcomplicated at this stage. In his opinion, the Partner States should continue with the current list as is by starting small and revising the list along the way. The organizer and facilitator of the meeting reassured the Partner States that this current list should be seen as a “living list” that will be adapted by stakeholders from each country during future expert working groups, according to the needs of the Partner States. In the end, the meeting participants found a middle ground by accepting the initial tracer list of products, supplemented with a brief list of hard-to-source products. [Fieldnotes Kigali meeting August 2023]

This excerpt provides an illustrative example of the complexity of alignment in practice. During this process, Partner States engage in negotiations that delve into their actual perceived problems. The complexity increases due to the diversity in characteristics among Partner States, leading to differences in their specific product needs. This negotiation is further shaped by the EAC’s general decision-making process. Unlike a majority-vote structure, decisions in the EAC are forged through consensus, granting each Partner State a theoretical veto power. However, the EAC’s strong hierarchical governance and accountability structure, which regional stakeholders are aware of, balances this dynamic. When higher-level officials have already decided on the way forward – in this case, implementing a pooled procurement mechanism – the efforts of regional stakeholders are primarily directed towards finding common ground and reaching agreements to realise that goal. Sometimes in the form of unanimity and, on other occasions, in the form of finding a middle ground, as demonstrated in the example above.

The stakeholders concluded the meeting with their approval to start information sharing (Phase 1), once the information-sharing platform had been developed and/or adapted. Finally, the meeting recommended the EAC Secretariat and the EAC RCE-VIHSCM to convene additional Expert Working Group meetings on legal, finance and procurement matters to develop appropriate implementation documents and guidelines as also proposed during the validation meeting in Entebbe.

At the time of writing, these additional working groups are being planned and conducted. The alignment process between Partner States is still in progress and it remains unknown when the EAC pooled procurement mechanism becomes operational, starting with information sharing.

## Discussion

Attempts to implement the EAC pooled procurement mechanism have been ongoing for nearly two decades. Despite intensified efforts in the past couple of years, the mechanism has not reached fruition yet. In this article, we set out to explore how the EAC pooled procurement has been developing over time and what work and efforts have been made to align inter-country interests. In-depth case studies of such collaboration initiatives are often lacking in academic literature. We believe that insights from this long-term study are relevant as they help to delineate complexities involved in setting up such collaboration initiatives in practice. In addition, the application of the Pooled Procurement Guidance as an analytic tool increases our understanding of why, how and when certain elements are essential during the developmental process.

So, why did the EAC pooled procurement mechanism originally stagnate? Our findings showed that a lack of dedicated funding, sufficient resources and ownership played an outsized role in the promise stage of the EAC pooled procurement mechanism. Following the initial multi-level engagement efforts between Partner States in the mid-2000s, the EAC Partner States started their pooled procurement journey with an attempt to align interests. After conducting a comprehensive needs assessment, the Partner States agreed on group contracting as the pooled procurement model for the region as early as 2008. In the years that followed, however, progress came to a halt due to a lack of dedicated funding and the absence of a designated secretariat to spearhead inter-country alignment and implementation processes going forward. Although progress was not always linear during this promise stage, incremental developments allowed the project to regularly recur on the EAC agenda.

After nearly a decade of dormancy, what prompted the EAC Partner States to pursue another attempt following the initial stagnation? Primarily, the establishment of a dedicated secretariat (EAC RCE-VIHSCM), whose main project was to take ownership and responsibility over the development of the pooled procurement mechanism through *alignment work* consisting of converging needs, managing the process and building inter-personal relationships. Since then, Partner States have been involved in an intricate dance of negotiations and harmonisation efforts led by the EAC Secretariat and the EAC RCE-VIHSCM. As our excerpts illustrate, this has been a continuous and reflexive process of negotiations and re-negotiations on challenges, needs and design during market survey analyses, feasibility studies and consecutive regional stakeholder meetings. In addition, our findings show that external experts (i.e. stakeholders from outside the EAC Partner States) play an important role in making sensitive issues manageable and debatable.

Going forward, however, we argue that continuous alignment efforts will be needed as this construct does not inherently sustain itself. Alignment remains fragile particularly due to the voluntary nature of participation and the availability of alternative venues (i.e. outside the pooled procurement mechanism) for participating countries to achieve their aims. In addition, sustained political will and long-term dedication of funding of Partner States will be required. This commitment will depend on the Partner States’ perception of equal representation, active involvement and fair allocation of benefits within the mechanism. In addition, strengthening inter-personal relationships among Partner States, as well as with the (yet to be established) procurement secretariat, will be essential for its success. Factors that can influence this relationship include continuous and broad involvement, mutual trust, absence of conflict of interest, history of collaboration, credibility and development of a positive reputation and track record (Parmaksiz, Bal, et al., 2023; Parmaksiz, van de Bovenkamp, et al., 2023). See Supplemental Material 1 for an in-depth analysis of these factors in the EAC pooled procurement mechanism. To reinforce these interpersonal dynamics, secure sustained funding, and acquire political buy-in, we suggest Partner States to continue the phased approach towards group contracting by starting with information sharing. This gradual and iterative process will facilitate learning from experience, while interim benefits, such as costs savings and increased availability, can further encourage commitment from Partner States.

Similar to the pooled procurement mechanism, the EAC has also been evolving. In the last two years, the Democratic Republic of the Congo and the Federal Republic of Somalia have both joined the EAC, but have not yet been actively participating in the development of the mechanism. Incorporating their needs into the pooled procurement mechanism can be viewed as both an opportunity and a challenge. While expansion increases the pool of regional experts, market size – and with that presumably buying power – and regional manufacturing capacity, it also requires more intensive alignment efforts and integration of in-country processes such as procurement and supply chain systems, financial operations and legal frameworks. Similarly, it is essential to consider and align the interests and needs of Partner States that are concurrently involved in other inter-country pooled procurement initiatives. The extent to which this alignment succeeds will be an important determinant for the mechanism’s sustainability.

### Strengths and limitations

Our (partial) involvement in the development of the EAC pooled procurement processes poses some risk of bias. To minimise this risk, we verified and triangulated our data in two ways. First, we used various data sources such as grey literature documents (e.g. policy papers, feasibility studies, newspaper articles), document analysis of regional stakeholder and ministerial meetings, observations and informal interviews with experts involved in earlier stages of the mechanism. Second, the involvement of experts from diverse backgrounds and countries has enabled us to verify and frequently reflect on our data and assumptions within our research team.

On the flip side, our close proximity to and involvement in these processes has provided a unique insight into the development of complex inter-country pooled procurement mechanisms. It has allowed us to observe, document and reflect on the continuous work and efforts of participating actors – including ourselves – over a longer period of time in terms of *alignment work*, consisting of facilitating negotiations, process management and inter-personal relationship building. We believe these detailed insights provide valuable academic and practical lessons for researchers, policy-makers, and other stakeholders by shedding light on the (frequently overlooked or undocumented) complexities involved in setting up such (inter-country) pooled procurement mechanisms.

## Conclusion

Our study provides insights in the longitudinal development of the EAC pooled procurement mechanism. It shows that setting up collaboration initiatives such as inter-country pooled procurement mechanisms require: (1) continuous consensus-building and alignment work to harmonise goals, needs and operations; (2) a driving force to lead this alignment work by taking ownership over the process and be receptive to the needs of stakeholders; and (3) fostering inter-personal relationship and trust among participants.

Moving forward, we believe that continuous alignment efforts, long-term political determination and sustained funding will be necessary to successfully achieve the gradual implementation of the EAC pooled procurement mechanism. To support this process, we recommend Partner States to regularly revisit the essential elements in the Pooled Procurement Guidance and reflect on their adoption and relevance as their needs and the mechanism evolves over time.

## Supplementary Material

Supplementary Material 1 - EAC Pooled Procurement Guidance

## Data Availability

Most data in this study are available in the public domain. Some internal reports are available from the corresponding author on reasonable request.
