# Supplementary material for: Towards regional access to medicines: the development of the East African Community pooled procurement mechanism
Source: J Pharm Policy Pract. 2024 Aug 21;17(1):2390653. doi: 10.1080/20523211.2024.2390653 (PMC11340228; doi:10.1080/20523211.2024.2390653)
Supplement: Supplementary Material 1 - EAC Pooled Procurement Guidance [file JPPP_A_2390653_SM3949.docx]

## Creation stage – East African Community (EAC)

## General characteristics and historical developments

The East African Community (EAC) is one of the eight Regional Economic Communities (RECs) recognised by the African Union. It was re-established in 2000 and consist of eight Partner States: The Democratic Republic of the Congo, the Republic of Burundi, the Republic of Kenya, the Republic of Rwanda, the Republic of South Sudan, the Republic of Uganda, the United Republic of Tanzania and the Federal Republic of Somalia. Currently, six out of eight Partner States are actively participating in setting up an inter-country pooled procurement mechanism. Because the Democratic Republic of the Congo and the Federal Republic of Somalia have only recently joined the EAC, they have not been actively participating in the development of the mechanism at this stage. Table 1 shows the demographic, geographic and economic characteristics of the participating EAC Partner States.

|  | Area/km^2^ | Population  (2023 est.) | GDP, PPP (current international $) (Millions) | GDP per capita, PPP (current international $) | Current health expenditure  (% of GDP) | Share of locally produced medicines (% of market) |
| --- | --- | --- | --- | --- | --- | --- |
| **Burundi** | 27,830 | 13,162,952 | 10,778.10 | 836.2 | 6.50 | 3 |
| **Kenya** | 580,367 | 57,052,004 | 311,409.58 | 5,763.9 | 4.29 | 30 |
| **Rwanda** | 26,338 | 13,400,541 | 38,470.35 | 2,792.4 | 7.32 | <1 |
| **South Sudan** | 644,329 | 12,118,379 | 13,230.90 | 1,181.9 | 5.25 | 0 |
| **Tanzania** | 947,300 | 65,642,682 | 196,630.46 | 3,096.9 | 3.75 | 12 |
| **Uganda** | 241,038 | 47,729,952 | 127,281.65 | 2,693.8 | 3.96 | 20 |
| **Somalia** | 637,657 | 12,693,796 | 24,004.79 | 1,364.1 | - | - |
| **DRC** | 2,344,858 | 111,859,928 | 132,415.38 | 1,337.4 | 4.05 | - |

**Table 1**. Demographic and Economic Characteristics of EAC Partner States. *Sources*: CIA World Factbook, World Bank (World Bank, n.d.-c, n.d.-b, n.d.-a), East African Community (East African Community, 2018a).

Since its inception, the EAC has implemented a customs union in 2005 and a common market in 2010 (East African Community, n.d.-a). Partner States have also made coordinated efforts to increase cooperation and integration in the health sector. Examples of such initiatives are the Mobile Laboratories project aimed at rapidly diagnosing and responding to communicable diseases in the region (Affara et al., 2021) and the EAC Medicines Regulatory Harmonization (MRH) project aimed at increasing access to quality medicines through collaboration and standardization between regulatory authorities (Mashingia et al., 2020; Sillo et al., 2020).

The EAC is governed by the following bodies (East African Community, n.d.-b):

- **The Summit**: comprising of the Heads of State of each Partner State. The Summit meets at least once a year and is responsible for providing strategic direction.
- **The Council of Ministers (‘The Council)**: comprising of Ministers or Cabinet Secretaries from the Partner States responsible for regional co-operation or EAC affairs. The Council of Ministers meets twice a year and is the central decision-making and governing organ of the EAC.
- **Coordinating Committee:** it is mainly responsible for facilitating regional co-operation and it coordinates the activities of the Sectoral Committees.
- **Sectoral Committees:** are proposed by the Coordinating Committee and formed by the Council of Ministers. These Sectoral Committees, such as the Sectoral Council of Ministers of Health, are responsible for conceptualising programmes and monitoring their implementation.
- **EAC Secretariat**: the executive Organ of the EAC. It is headquartered in Arusha, Tanzania and its staff members are responsible for carrying out the daily work as mandated by the Council.
- **East African Legislative Assembly (EALA):** is the legislative body of the EAC responsible for legislation, provision of oversight and representation of the East African people. The EALA consists of 72 members: 63 elected members (9 from each Partner State) elected by their respective country's national legislature, 9 ex officio members consisting of the Minister or Cabinet Secretary responsible for EAC Affairs from each Partner State, the Secretary-General and the Counsel to the Community.
- **East African Court of Justice**: is the principal judicial organ of the EAC and ensures adherence to the law in the interpretation and application of compliance with the EAC Treaty. The Court is composed of ten judges, appointed by the Summit.

The data in the following table is based on literature review, document analysis of the EAC Sectoral Council of Ministers of Health meeting reports, observations during various EAC Regional Stakeholder meetings and data collected for the purpose of the East African Community Pooled Procurement of Medicines and Health Commodities Market Survey Report.

| **Essential elements/actor** | ***East African Community (EAC)*** | **References** |
| --- | --- | --- |
| 1. **Buyers** |  |  |
| **All buyers need to have individually**: | |  |
| 1. Perceived problem for which pooled procurement may be a solution (problem) | EAC Partner States have been experiencing various problems limiting their access to affordable and quality-assured medicines. These includes:   - Insufficient market volumes - High prices for essential medicines - Shortages of essential medicines - Limited market size and production capacity for local manufacturers - Unreliable supplier participation in tenders - Uncertain/low quality of certain products - Fragmentation of procurement and supply chain processes - Inaccurate demand forecasting data | (East African Community, 2018a; WHO et al., 2007; Yenet et al., 2023) |
| 1. Motivations that outweigh the opportunity costs | EAC Partner States have a variety of motivations to participate in the pooled procurement mechanism:   - Increase economies of scale for certain health products - Incentivize and attract suppliers in tenders - Availability of medicines at affordable prices - Increased sharing of information and expertise - Inter-country procurement capacity building - Improve data quality (e.g., demand planning, prices, supplier performance, etc.)   The potential downsides of participation include:   - Reduced flexibility in procurement processes - Additional investment needed to train staff - Added bureaucracy - Misalignment of different procurement legal frameworks - Existing and ongoing procurement arrangements with suppliers - Differences in local manufacturing capacities between Partner States |  |
| 1. Budget, either internal or external (through donors) | There are differences between EAC Partner States in how medicines are financed:   - Most Partner States have their own capital at their respective CMS. Therefore, they are likely to have immediate access to funding to pay suppliers. - Other Partner States receive funding from the Ministry of Finance on a monthly or quarterly basis and may or may not have immediate access to funding. - Some Partner States have their procurement outsourced and managed to a Procurement Service Agent. |  |
| 1. Sufficient technical capacity (e.g., demand forecasting) | Most Partner States generate annual yearly forecasts based on previous year’s consumption data with approximately the same time frame. However, accuracy of data at the CMS is sometimes affected by inaccurate or incomplete consumption data from the health facilities*.*  In addition, some Partner States experience a lack of experienced and dedicated staff to carry out the demand forecasting and procurement at the health facility level*.* As a result, health professionals such as doctors, nurses and clinical officers have been tasked to carry out procurement activities, in addition to their existing duties. | (Mackintosh et al., 2018; WHO et al., 2007) |
| 1. Compatible laws, regulations and policies that allow for (international) pooled procurement | According to a 2007 feasibility study, most EAC Partner States share a common procurement legislation, policy and institutional framework based on the United Nations Commission on International Trade Law (UNCITRAL). Most Partner States did not expect to legislation to be a barrier for inter-country pooled procurement. One of the Partner States believed at that time that harmonizing policies might pose a problem. Therefore, harmonization of procurement legislation was recommended.  A 2017 feasibility study reaffirmed the need to harmonize procurement legislation among EAC Partner States. This harmonized legislation should “build on the common elements in the national procurement legislations of EAC Partner States.”  However, the current status of legislation on inter-country pooled procurement is unknown and requires further study. Based on data collected during the *Regional Stakeholders’ Meeting to Build Consensus on the Procurement Model for the EAC Partner States* in Nairobi, Kenya in 2020, 3 out of 6 EAC Partner States do not specifically recognize inter-country pooled procurement in their national legislation or regulation. Therefore, it is likely that national regulations or legislations have to be adapted and harmonized before pooled procurement can be implemented in these EAC Partner States.  To incentivize national production, create jobs and increase supply security, most EAC Partner States have a national preference policy for local manufacturers in place, providing national manufacturers a 15% price advantage over international suppliers. | (East African Community, 2017b; WHO et al., 2007) |
| **If buyer's mechanism, all buyers combined, need to have:** | |  |
| 1. Demonstrated willingness to solve their problem collectively through pooled procurement (shared vision) | Numerous high-level meetings have been held on pooled procurement in the EAC since its inception. Some of the more influential ones in the initial years have been listed below:   - In 2005, the 1^st^ Ordinary Meeting of the Sectoral Council of the Ministers of Health recommended to carry out a situational analysis study on “medicines policy, legal and regulatory framework, procurement, distribution and management within the EAC Partner States to facilitate the process of harmonisation within the EAC”. - In 2007, the health ministers considered and approved the final report and recommendations of the situational analysis during the 2^nd^ Ordinary Meeting of the Sectoral Council of Ministers of Health. - In March 2008, the 15th EAC Council of Ministers approved the “Regional Group-Contracting Pooled Bulk Procurement Model” for Priority Essential Medicines in East Africa. - In September 2008, the 3^rd^ Ordinary Meeting of the Sectoral Council of Ministers of Health directed the EAC Secretariat to develop a draft strategic plan of action and multi-year budget for the implementation of pooled procurement. In addition, the *Regional Essential Medicines Pooled Bulk Procurement Expert Task Force* was established with a wide variety of stakeholders appointed by the health ministers. - During the 5^th^ Ordinary Meeting of the Sectoral Council of Ministers of Health in 2011, the health ministers reiterated their directive to the EAC Secretariat to finalize the development of a business plan and prepare the Terms of Reference (TORs) for the National Task Forces in the EAC Partner States.   In the following years, limited progress was made on pooled procurement, until around 2016:   - The topic of pooled procurement was not discussed during three consecutive Ordinary Meeting of the Sectoral Council of Ministers of Health, between 2011-2012. - During the 9^th^ Ordinary Meeting of the Sectoral Council of Ministers of Health in April 2014, the EAC Sectoral Council of Ministers of Health directed the EAC Secretariat to set up Regional Centres of Excellence to stimulate progress and increase ownership of various priority areas in the region. - In March 2016, the EAC Regional Centre of Excellence for Vaccines, Immunization, and Health Supply Chain Management (EAC RCE-VIHSCM) was established. One of its responsibilities was to drive the implementation of the inter-country pooled procurement of health commodities in the EAC region in collaboration with the EAC Secretariat.   In more recent years, the EAC Partner States have reaffirmed their political will to implement an inter-country pooled procurement mechanism by taking a stepwise approach.   - In November 2019, the Ministers of Health directed the EAC Secretariat to coordinate additional in-country consultations on the pooled procurement model to be adopted during the 19^th^ EAC Sectoral Council of Ministers of Health Meeting. - In April 2021, the Ministers of Health adopted the proposed group contracting model and the detailed roadmap towards pooled procurement in the EAC during the 20th EAC Sectoral Council of Ministers of Health Meeting. - In December 2021, the Ministers of Health took note of the progress made in the development of the EAC Pooled Procurement Framework and directed the EAC Secretariat to officially request Partner States to submit the top 5 categories of health commodities to be included in the pooled procurement mechanism during the 20th EAC Sectoral Council of Ministers of Health Meeting. | (East African Community, 2005, 2007, 2008, 2011a, 2011b, 2012a, 2012b, 2014, 2017a, 2019, 2021a, 2021b; WHO et al., 2007) |
| 1. Alignment on goals, purpose and operations of the pooled procurement mechanism (shared plan) | Various regional stakeholder meetings have been held among EAC Partner States to reach alignment on goals, purpose and operations of the pooled procurement mechanism. Some of the more prominent ones have been listed below:   - In 2006, the EAC regional stakeholders recommended during a meeting Zanzibar to further develop the possibility of pooled procurement of antiretroviral (ARV) medicines, and also to ensure that the agenda of Regional Pooled Procurement of Medicines in the East African Community pooled procurement will continue to remain a high health care priority in the EAC. The meeting also agreed that the Group Contracting model of procurement should be a potential medium-term goal for the EAC. - In 2007, this situational analysis was carried out by the Technical Cooperation for Essential and Traditional Medicines (TCM) department of the WHO in collaboration with Management Sciences for Health (MSH) and John Snow Incorporate (JSI). This comprehensive assessment looked at political and organizational commitment, procurement legislations and policies, medicines regulatory procedures, medicine supply chain systems, financial resources & systems, and pricing policies. Based on their findings, the consultants suggested that group contracting would be more feasible for the EAC than central contracting - In September 2009, stakeholders convened during a regional meeting on *Regional Harmonisation of Policies, Legal and Regulatory Framework for EAC Partners States National Medicines Regulatory Authorities (NMRAs) and National Medicines Procurement Agencies (NMPAs)*. During plenary discussions, the stakeholders agreed to tackle potential issues of resistance to change among NMPAs, to develop a regional Essential Medicine List, to prioritize activities to ensure progress, and to update the members of the Expert Task Force.   In recent years, the number of regional stakeholder meetings have increased. These meetings have been facilitated by the EAC Secretariat and the EAC RCE-VIHSCM:   - In 2017, IMS Health, EAC RCE-VIHSCM and UNFPA conducted a feasibility study and situational analysis on cross-border supply chain solutions for reproductive health - In 2018, the outcomes of the feasibility study and situational analysis on cross-border supply chain solutions for reproductive health were discussed during a stakeholder meeting in Naivasha, Kenya. - In 2020, the *Regional Stakeholders’ Meeting to Build Consensus on the Procurement Model for the EAC Partner States* was held in Nairobi, Kenya. - In 2021, the experts from Partner States met again during *Regional Meeting to Develop a Detailed Model and Operational Plan for Pooled Procurement* was held in Nairobi, Kenya. - In March 2023, the *Regional Meeting to Validate the EAC Pooled Procurement Market Survey Report and Model* was held in Entebbe, Uganda. - In August 2023, CEOs of the National/Central Medical Stores from each Partner State convened in Kigali, Rwanda to discuss key findings of the 2023 Market Survey Report, the proposed governance structures and the information to be shared. The main goal was to update and obtain buy-in from National/Central Medical Stores. | (East African Community, 2009, 2017b, 2018b, 2020, 2021c, 2023b, 2023a; WHO et al., 2007) |
| 1. Joint need for specific products (product alignment) | The EAC Partner States have agreed to focus on the pooled procurement of essential medicines. In December 2021, the Ministers of Health directed the EAC Secretariat to officially request Partner States to submit the top 5 categories of health commodities to be included in the pooled procurement mechanism during the 20^th^ EAC Sectoral Council of Ministers of Health Meeting.  Currently, the EAC Partner States are in the process of identifying specific products to procure collaboratively. As a first step, the EAC Partner States agreed not to focus on donated products such as anti-malarials and anti-Retroviral (ARV) medicines. In addition, Partner States are also considering and discussing to include *hard to source* products in the pooled procurement mechanism. All Partner States also agreed that inter-country pooled procurement should prioritize and benefit local manufacturers in the region. | (East African Community, 2021a, 2023b) |
| 1. Sufficient market size | Based on 2023 population estimates in Table 1, the total population of the 6 actively participating EAC Partner States in the pooled procurement mechanism is estimated to be 209 million. This number can be potentially increase up to 330 million when DRC and Somalia participate in the pooled procurement mechanism in future years. Therefore, EAC Partner States have a sufficient potential market size in terms of volume. | (CIA, n.d.) |
| 1. Sufficient and stable financial capacity | The funding structures of most EAC Partner States are facilitative for inter-country pooled procurement. Other EAC Partner States might benefit from the following mitigation strategies to bring their funding structures closer to an optimal situation for inter-country pooled procurement:   - Advocate for independent operational capital for the procurement of health products at the Central Medical Stores, as mentioned under A3; - Engage the Ministries of Finance (MoF) in the implementation process of the mechanism in to secure political will and commitment. |  |
| 1. Regulatory harmonization (e.g., shared quality standards, joint assessment, market authorization, etc.) | The East African Community Medicines Regulatory Harmonization (EAC-MRH) Programme was established in 2012 with the aim to increase access to quality medicines. In 2015, the EAC Joint Assessment Program was launched, reducing the average drug approval time from 24 to 12 months. In 2016, the EAC-MRH project has started issuing GMP certificates centrally to manufacturers producing for the EAC market. Currently, medicines are still registered individually in each country, but the EAC-MRH’s goal is to achieve mutual recognitions among Partner States.  The EAC-MRH program is a great potential facilitator for the inter-country pooled procurement mechanism. For the pooled procurement mechanism to succeed, it is imperative that the future pooled procurement secretariat (PPS) and the EAC-MRH program closely collaborate. The EAC PPS can determine priority health commodities that require market authorization, while the EAC-MRH program can offer insights in the products or manufacturers that have already undergone joint assessment. In future, adopting a central registration system for health commodities in the EAC-MRH would further expedite the pooled procurement process. | (Mashingia et al., 2020; Sillo et al., 2020) |
| 1. Trust (in other buyers and the pooled procurement organization) | The regular regional stakeholder meetings in recent years, as described under A6, have contributed to increased levels of trust between the Partner States. Continuity in representation of stakeholders has also facilitated this, since stakeholders have also been able to intensify personal relationships. In addition, the presence of an independent third-party organization (i.e., EAC RCE-VIHSCM) that initiates engagement, facilitates meetings and drives the process forward contributes to trust-building between key actors.  To maintain or strengthen trust levels, however, several other factors are essential between Partner States during the creation process. These include:   - Transparent data and information sharing between Partner States, as described under A13; - Continued alignment on goals, product needs, purpose and operations of the pooled procurement mechanism, as described under A7&A8. - Setting up a procurement secretariat with clear roles and responsibilities, and operations based on a collectively shared operational manual with standardized procedures; - Setting up a predictable, timely and efficient payment mechanism that is accepted by all Partner States. |  |
| 1. Transparent data and information sharing | All Partner States make use of an IT system for national procurement. However, these systems differ between the Partner States. As part of their stepwise approach to implement inter-country pooled procurement, the EAC Partner States are currently in the process of identifying existing information sharing platforms that can facilitate transparent data and information sharing on medicine prices, product quality and supplier performances. During multiple meetings in 2023 and 2024, EAC Partner States have been discussing which type of information/data to share and which formats to use during Phase 1 of the pooled procurement mechanism (i.e., information sharing). |  |
| 1. No history of conflict or failed collaboration | The EAC Partner States have a long history of cooperation. In 1917, Kenya and Uganda formed a Customs Union, which Tanganyika (now the United Republic of Tanzania) joined in 1927. Between 1948-1961 these three countries formed the East African High Commission (EAHC), which was renamed after independence of the countries into East African Common Services Organisation (EACSO) between 1961-1967, and later into the East African Community (EAC) in 1967 with the aim to achieve economic growth within the region by adopting policies such as creating a common market and a common customs tariff. Although these policies were not achieved, the EAC managed to establish public enterprises such as the East African Railways and Harbours, East African Airways, East African Posts and Telecommunications and East African Development Bank. After political unrest in the 1970s in the region, the East African Community was dissolved in 1977. In 2000, the EAC was re-established, as explained under A17. | (Cooksey, 2016; East African Community, n.d.-a, n.d.-c) |
| 1. Homogeneity of buyer's characteristics related to their needs | In general, the EAC Partner States face relatively similar challenges with regard to procurement of essential medicines, as described under A1. Therefore, the motivations to participate seem relatively convergent among Partner. In terms of demographic and economic characteristics, there are some differences between Partner States (see Table 1). The levels of technical capacity, however, are relatively similar among most of the Partner States. |  |
| 1. Shared cultural factors and values (e.g., language, traditions, etc.) | The EAC Partner States share many commonalities in history, language and culture. Currently, the official language of the EAC is English. However, discussion have been ongoing for the past couple of years to add French and Kiswahili as official languages. | (Leonard A. Kamwanja et al., 2010) |
| 1. Existing political or structural mechanisms | After the *Treaty for the Establishment of the East African Community*was signed in November 1999 and came into force in July 2000, Kenya, Uganda and Tanzania became the three founding Partner States of the revived EAC. Rwanda and Burundi joined the EAC in 2007, South Sudan in 2016, the Democratic Republic of Congo (DRC) in 2022 and Somalia in 2023. Since initiation, the EAC has implemented a Customs Union (2005) and a Common Market (2010). EAC’s next goals are setting up a Monetary Union and a Political Federation.  The EAC has a well-organized and hierarchical governance structure:   - At the top, there is the *Summit*, which consists of State of each Partner State. - One level below is the *Council of Ministers*, comprising of Ministers or Cabinet Secretaries from the Partner States responsible for regional co-operation or EAC affairs. It is the central decision-making and governing organ of the EAC. - Under the Council of Ministers, there is the *Coordinating Committee*, responsible for facilitating regional cooperation and coordination of activities, and the *Sectoral Committees*, who are responsible for conceptualizing programs and monitoring their implementation. Finally, there is the *EAC Secretariat*, which is the executive organ of the EAC, headquartered in Arusha, Tanzania. - In addition, the *East African Legislative Assembly* (EALA) is responsible for legislation, provision of oversight and representation of the East African people, while the *East African Court of Justice* is the principal judicial organ of the EAC and ensures adherence to the law in the interpretation and application of compliance with the EAC Treaty. The Court is composed of ten judges, appointed by the Summit.   Furthermore, most of the EAC Partner States are also part of other Regional Economic Communities (RECs) in Africa. This might potentially complicate harmonization and alignment processes within the EAC, if rules and regulations are conflicting with other RECs. In addition to the EAC, Partner States are members of:   - Tanzania is a member of SADC; - Uganda is a member of IGAD and COMESA; - Kenya is a member of IGAD, COMESA and CEN-SAD; - South Sudan is a member of IGAD; - Burundi and Rwanda are members of COMESA and ECCAS; - DRC is a member of COMESA, ECCAS and SADC; - Somalia is a member of IGAD and CEN-SAD. | (East African Community, n.d.-a, n.d.-c; ECDPM, n.d.; East African Community, 2002) |
| 1. **Pooled procurement organization** |  |  |
| 1. Organizational structure with clear roles and responsibilities |  |  |
| 1. Clear mandate |  |  |
| 1. Standardized and transparent procedures |  |  |
| 1. Sufficient, predictable and timely budget, either internal (through service fees) or external (through donors) to carry out pooled procurement |  |  |
| 1. Sufficient, predictable and timely budget, either internal (through service fees) or external (through donors), to cover organizational expenses |  |  |
| 1. Predictable, timely and efficient payment mechanism |  |  |
| 1. Human resources (sufficient in numbers and expertise) |  |  |
| 1. Sufficient technical capacity (e.g., procurement, quality assessment, forecasting, etc.) |  |  |
| 1. Positive reputation |  |  |
| 1. No conflict of interest |  |  |
| 1. User-friendliness (both towards buyers and sellers) |  |  |
| 1. **Suppliers** |  |  |
| 1. Sufficient number of qualified suppliers | One of the challenges that EAC Partner States experience is unreliable supplier participation in national tenders, as mentioned under A1. In addition, Partner States have significant differences in the presence and size of a domestic manufacturing industry. | (WHO et al., 2007) |
| 1. Sufficient production incentives for international and domestic manufacturers | The EAC provides several production incentives to local manufacturers, such as:   - Sufficient market size in terms of volume - Tax deduction and price advantages for local manufacturers over international manufacturers, as described under A5. | (WHO et al., 2007) |
| 1. Sufficient supply incentives | The EAC provides several supply incentives to manufacturers, such as:   - EAC-MRH Regulatory harmonization program that provides market access to the region - Sufficient market size in terms of volume - Long-term framework agreements   Additional suggestions to increase supply incentives include:   - Adopt a predictable, timely and efficient payment mechanism for suppliers - Agree on unified packaging labels - Develop standardized and transparent procurement manuals - Improve accuracy of demand forecasting data | (Mashingia et al., 2020; Sillo et al., 2020) |
| 1. Sufficient number of distributors with favourable delivery terms | One important area that needs to be agreed upon among EAC Partner States are accepted incoterms. The geography of the Partner States differs greatly. While some Partner States are landlocked countries, others have access to sea ports. This means that it is generally more expensive for a supplier to offer products to landlocked countries. The presence of infrastructure such as large international airports that can serve as a regional hub is also beneficial for securing favorable delivery terms and selecting appropriate incoterms. | (WHO et al., 2007) |

## References

Affara, M., Lagu, H. I., Achol, E., Karamagi, R., Omari, N., Ochido, G., Kezakarayagwa, E., Kabatesi, F., Nkeshimana, A., Roba, A., Ndia, M. N., Abudo, M. U., Kabanda, A., Mpabuka, E., Mwikarago, E. I., Kutjok, P. E., Samson, D. D., Deng, L. L., Moremi, N., … Gehre, F. (2021). The East African Community (EAC) mobile laboratory networks in Kenya, Burundi, Tanzania, Rwanda, Uganda, and South Sudan—From project implementation to outbreak response against Dengue, Ebola, COVID-19, and epidemic-prone diseases. *BMC Medicine*, *19*(1), 160. https://doi.org/10.1186/s12916-021-02028-y

CIA. (n.d.). *Country Comparisons—Population*. The World Factbook. Retrieved from: https://www.cia.gov/the-world-factbook/field/population/country-comparison/

Cooksey, B. (2016). *Tanzania and the East African Community: A comparative political economy* (186; Discussion Paper). European Centre for Development Policy Management. https://ecdpm.org/wp-content/uploads/ECDPM-Discussion-Paper-186-Tanzania-East-African-Community-Comparative-Political-Economy-2016.pdf

East African Community. (n.d.-a). *EAC History*. Retrieved from: https://www.eac.int/eac-history

East African Community. (n.d.-b). *EAC Organs*. Retrieved from: https://www.eac.int/eac-organs

East African Community. (n.d.-c). *EAC Quick Facts*. Retrieved from: https://www.eac.int/eac-quick-facts

East African Community. (2002). *The Treaty for the Establishment of the East African Community*. https://www.eac.int/index.php?option=com_documentmananger&task=download.document&file=bWFpbl9kb2N1bWVudHNfcGRmX0RpcEV6WXRITVRreWhsRVFicXNmVkRNRUFDIFRSRUFUWQ==&counter=13

East African Community. (2005). *1st Ordinary Meeting of the EAC Sectoral Council of Ministers of Health* (EAC/SC/01/2005).

East African Community. (2007). *2nd Ordinary Meeting of the EAC Sectoral Council of Ministers of Health* (EAC/SR/30/2007).

East African Community. (2008). *3rd Ordinary Meeting of the EAC Sectoral Council of Ministers of Health* (EAC/MS/01/2008).

East African Community. (2009). *Meeting on Regional Harmonisation of Policies, Legal and Regulatory Framework for EAC Partner States National Medicines Regulatory Authorities (NMRAs) and National Medicines Procurement Agencies (NMPAs)* (EAC/TF/91/2009).

East African Community. (2011a). *5th Ordinary Meeting of the EAC Sectoral Council of Ministers of Health* (EAC/SCM/08/2011).

East African Community. (2011b). *6th Ordinary Meeting of the EAC Sectoral Council of Ministers of Health* (EAC/SC/21/2011).

East African Community. (2012a). *7th Ordinary Meeting of the EAC Sectoral Council of Ministers of Health* (EAC/SC/149/2012).

East African Community. (2012b). *8th Ordinary Meeting of the EAC Sectoral Council of Ministers of Health* (EAC/SC/157/2012).

East African Community. (2014). *9th Ordinary Meeting of the EAC Sectoral Council of Ministers of Health* (EAC/SCM/HEALTH/001/2014).

East African Community. (2017a). *15th Ordinary Meeting of the EAC Sectoral Council of Ministers of Health* (EAC/HEALTH/SCM15/2017).

East African Community. (2017b). *UNFPA - RCE VIHSCM Cross-Border Supply Chain for Reproductive Health Commodities*.

East African Community. (2018a). *2nd EAC Regional Pharmaceutical Manufacturing Plan of Action 2017-2027*.

East African Community. (2018b). *Regional Meeting to Validate the Draft Report of the EAC Cross-Border Situational Analysis and Feasibility Study on Reproductive Health Supply Chain Solutions and Pooled Bulk Procurement and Development of the Implementation Roadmap*.

East African Community. (2019). *19th Ordinary Meeting of the EAC Sectoral Council of Ministers of Health* (EAC/SCHEALTH/19/2019).

East African Community. (2020). *Regional Stakeholders’ Meeting to Build Consensus on the Pooled Procurement Model for the EAC Partner States*.

East African Community. (2021a). *20th Ordinary Meeting of the EAC Sectoral Council of Ministers of Health* (EAC/SCHEAL TH/20/2021).

East African Community. (2021b). *21st Ordinary Meeting of the EAC Sectoral Council of Ministers of Health*.

East African Community. (2021c). *Regional Meeting to Develop a Detailed Model and Operational Plan for Pooled Procurement*.

East African Community. (2023a). *Meeting of Heads/CEOs of the National (Central) Medical Stores*.

East African Community. (2023b). *Regional Meeting to Validate the EAC Pooled Procurement Market Survey Report and Model*.

ECDPM. (n.d.). *Regional Organisations in Africa*. Retrieved from: https://indd.adobe.com/view/f49ac87d-7aa3-4cf7-822e-841d674bbc92

Kamwanja L.A., Saka J., Awotedu A., Fute I., Chamdimba C., & Ndomondo-Sigonda, M. (2010). *Situation Analysis Study on Medicines Registration Harmonisation in Africa: Final Report for the East African Community (EAC)*. AUDA-NEPAD. https://www.nepad.org/file-download/download/public/15528

Mackintosh, M., Tibandebage, P., Karimi Njeru, M., Kariuki Kungu, J., Israel, C., & Mujinja, P. G. M. (2018). Rethinking health sector procurement as developmental linkages in East Africa. *Social Science & Medicine*, *200*, 182–189. https://doi.org/10.1016/j.socscimed.2018.01.008

Mashingia, J. H., Ahonkhai, V., Aineplan, N., Ambali, A., Angole, A., Arik, M., Azatyan, S., Baak, P., Bamenyekanye, E., Bizoza, A., Chamdimba, C., Doerr, P., Fimbo, A., Gisagara, A., Hamad, H., Harris, R., Hartman, D., Kabatende, J., Karangwa, C., … Mukanga, D. (2020). Eight years of the East African Community Medicines Regulatory Harmonization initiative: Implementation, progress, and lessons learned. *PLOS Medicine*, *17*(8), e1003134. https://doi.org/10.1371/journal.pmed.1003134

Sillo, H., Ambali, A., Azatyan, S., Chamdimba, C., Kaale, E., Kabatende, J., Lumpkin, M., Mashingia, J. H., Mukanga, D., Nyabenda, B., Sematiko, G., Sigonda, M., Simai, B., Siyoi, F., Sonoiya, S., Ward, M., & Ahonkhai, V. (2020). Coming together to improve access to medicines: The genesis of the East African Community’s Medicines Regulatory Harmonization initiative. *PLOS Medicine*, *17*(8), e1003133. https://doi.org/10.1371/journal.pmed.1003133

WHO, MSH, & JSI. (2007). *A Situational Analysis and Feasibility Study on Regional Pooled Bulk Procurement of Essential Medicines and Other Health Supplies in the East African Community Partner States*. http://repository.eac.int/bitstream/handle/11671/568/EAC%20Medicines%20Pooled%20Procurement%20Report%20-%202007.pdf?sequence=1&isAllowed=y

World Bank. (n.d.-a). *Current health expenditure (% of GDP)—Burundi, Kenya, Rwanda, South Sudan, Tanzania, Uganda, Congo, Dem. Rep., Somalia* [dataset]. Retrieved from: https://data.worldbank.org

World Bank. (n.d.-b). *GDP per capita, PPP (current international $)—Burundi, Kenya, Rwanda, South Sudan, Tanzania, Uganda, Congo, Dem. Rep., Somalia* [dataset]. Retrieved from: https://data.worldbank.org

World Bank. (n.d.-c). *GDP, PPP (current international $)—Burundi, Kenya, Rwanda, South Sudan, Tanzania, Uganda, Congo, Dem. Rep., Somalia* [dataset]. Retrieved from: https://data.worldbank.org

Yenet, A., Nibret, G., & Tegegne, B. A. (2023). Challenges to the Availability and Affordability of Essential Medicines in African Countries: A Scoping Review. *ClinicoEconomics and Outcomes Research: CEOR*, *15*, 443–458. https://doi.org/10.2147/CEOR.S413546
